# Diagnosis of Hourglass-Like Constriction Neuropathy of the Radial Nerve Using High-Resolution Magnetic Resonance Neurography: A Report of Two Cases

**DOI:** 10.3390/diagnostics10040232

**Published:** 2020-04-17

**Authors:** Du Hwan Kim, Duk Hyun Sung, Min Cheol Chang

**Affiliations:** 1 Department of Physical Medicine and Rehabilitation, College of Medicine, Chung-Ang University, Seoul 06973, Korea; ri-pheonix@hanmail.net; 2 Department of Physical Medicine and Rehabilitation, Samsung Medical Center, Sungkyunkwan University School of Medicine, Seoul 06351, Korea; yays.sung@samsung.com; 3 Department of Physical Medicine and Rehabilitation, College of Medicine, Yeungnam University, 317-1, Daemyungdong, Namku, Taegu 705-717, Korea

**Keywords:** hourglass-like constriction neuropathy, magnetic resonance neurography, radial nerve, ultrasound

## Abstract

Hourglass-like constriction neuropathy is a neurological condition caused by fascicular constriction of one or more peripheral nerves, unrelated to intrinsic or extrinsic compression. It is often neglected in clinical practice, and its diagnosis is challenging. Here, we report two cases of hourglass-like constriction neuropathy in the radial nerve diagnosed using high-resolution magnetic resonance neurography (MRN). Two men, aged 47 and 19 years, developed sudden weakness in the left wrist and finger extensors. They were diagnosed with radial neuropathy between the left mid-humerus level and the elbow joint, using the electrodiagnostic test. To evaluate the cause of the nerve lesion and the lesion location, high-resolution MRN was performed. Patient 1 showed an hourglass-like constriction of the left posterior interosseous nerve within the epineurium of the left radial nerve, 8.9 cm proximal to the lateral epicondyle. Patient 2 showed two focal constrictions of the left radial nerve, 8.0 and 6.9 cm proximal to the lateral epicondyle, respectively, and distal to the radial groove. Additionally, bull’s eye signs were observed juxta-proximal to constrictions of the left radial nerve. The findings were indicative of hourglass-like constriction neuropathy. Both of the patients underwent surgery. However, at the 6-month follow-up, their motor weakness showed no improvement. MRN can be beneficial for diagnosing hourglass-like constriction neuropathy and locating the lesion.

## 1. Introduction

Hourglass-like constriction neuropathy is a neurological condition caused by fascicular constriction of one or more peripheral nerves, unrelated to intrinsic or extrinsic compression or trauma [1,2,3]. Hourglass-like constriction neuropathy can manifest as sudden neurological symptoms, such as paralysis, sensory deficit and neuropathic pain [1,2,3]. No treatment protocol for hourglass-like constriction neuropathy has been established, but surgery has proven beneficial [3]. The accurate diagnosis of hourglass-like constriction neuropathy is challenging, as it is not observed in conventional magnetic resonance imaging (MRI) and is usually detected during exploratory surgery [1].

Recently developed high-resolution magnetic resonance neurography (MRN) provides high-resolution images of the peripheral nerves by suppressing both fat and vessel signals [4,5,6]. The bull’s eye sign is a specific finding of hourglass-like constriction neuropathy [6]. It refers to the finding of peripheral and central hypointensity on fat-suppressed nerve axial imaging [6], and indicates focal edematous swelling of the nerve proximal to the constriction sites. The nerve-sheath signal increased with inked rest-tissue rapid acquisition of relaxation enhancement imaging (SHINKEI) sequence, which was initially introduced by Yoneyama et al. [7], which clearly demonstrates the brachial plexus, lumbosacral plexus, and cranial nerve. This sequence includes two parts: the fat-suppression pre-pulse and improved Motion Sensitized Driven Equilibrium (MSDE) pre-pulse to suppress vessels, followed by a readout section with a 3D, tissue-specific, variable refocusing, flip-angle rapid acquisition with relaxation enhancement (RARE) sequence to acquire contrast-efficient, T2-weighted images [7]. The SHINKEI sequence provides a better nerve-to-fat ratio, muscle-to-fat ratio, and nerve signal-to-noise and contrast-to noise ratios than the fat suppression pre-pulse alone [8]. This sequence can yield high-resolution and volumetric neurographic images by suppressing both the fat and vessel signals to detect torsion sites along the longitudinal axis of an individual branch from the brachial plexus [8]. The potential application of MRN in the detection of any morphologic changes in the individual nerve in sudden spontaneous paralysis has been reported [1,6,9]. However, little is known about the MRN findings of hourglass-like constriction neuropathy or its usefulness in obtaining the diagnosis.

Here, we report two cases of hourglass-like constriction neuropathy in the radial nerve, diagnosed using high-resolution MRN.

## 2. Case Presentation

### 2.1. Patient 1 

**Figure 1:**
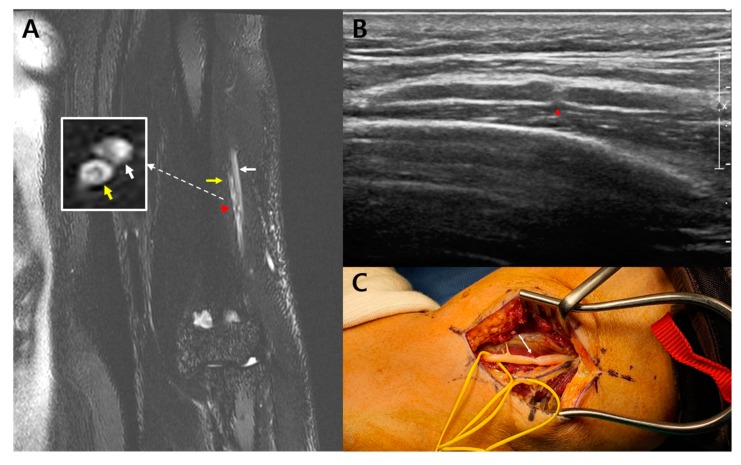
Case 1: (**A**) High-resolution magnetic resonance neurography reveals a focal constriction (red arrowhead) of the left posterior interosseous nerve (PIN) (yellow arrows) within the epineurium of the left radial nerve 8.9 cm proximal to the lateral epicondyle. The left superficial radial nerve (white arrows) is swollen. Further, the bull’s eye sign (yellow arrow in white square) is observed juxta-proximal to the constriction site of the left PIN; (**B**) Longitudinal ultrasound reveals constriction (red arrowhead) of the left radial nerve; (**C**) Intraoperative finding confirms constriction (write arrow) of the left radial nerve.

A 47-year-old man visited the department of physical medicine and rehabilitation at Samsung medical center because of a sudden onset of left wrist drop 3 weeks before presentation while working, with no identifiable cause. A day before the onset of the wrist drop, he had experienced elbow and posterior forearm pain that had persisted for a day. He had no relevant medical history. A physical examination revealed complete paralysis of the left wrist and finger extensors. No sensory deficits were observed. The patient experienced pain proximal to the elbow joint during active and passive range of motion. Approximately 3 weeks after the symptom onset, we performed cervical magnetic resonance imaging, electrodiagnostic study, ultrasound, and high-resolution MRN. The cervical MRI revealed no abnormalities. In the nerve conduction study (NCS), the compound muscle action potential (CMAP) of the left radial nerve showed a lower amplitude than that of the right radial nerve (left side: 0.9 mV vs. right side: 7.8 mV). On needle electromyography (EMG), positive sharp waves were generated in the left brachioradialis, extensor carpi radialis longus, extensor digitorum communis, and extensor indicis muscles. These muscles showed no motor unit action potential (MUAP) during volitional activity. The NCS and EMG findings indicated radial neuropathy between the left mid-humerus level and the elbow joint. To obtain the definitive diagnosis and determine the exact lesion location, a high-resolution 3-Tesla MRN, including the SHINKEI sequence, was performed. The high-resolution MRN revealed a constriction of the left posterior interosseous nerve within the epineurium of the left radial nerve, 8.9 cm proximal to the lateral epicondyle, and distal to the radial groove (Figure 1A). Further, the bull’s eye sign was observed juxta-proximal to the constriction site of the left radial nerve. A longitudinal ultrasound revealed nerve constriction in the area corresponding to the constriction observed on MRN (Figure 1B). The patient was treated with intravenous steroids (methylprednisolone 500 mg for 3 days) and tapering of oral prednisolone. Three months after symptom onset, the patient’s motor function showed no improvement, and he underwent end-to-end neurorrhaphy. The surgical findings revealed the precise location of constriction of the left radial nerve identified on MRN (Figure 1C). No muscle or other soft tissue causing nerve compression was observed. At the 6-month postoperative follow-up, the patient’s motor weakness showed no improvement.

### 2.2. Patient 2

**Figure 2:**
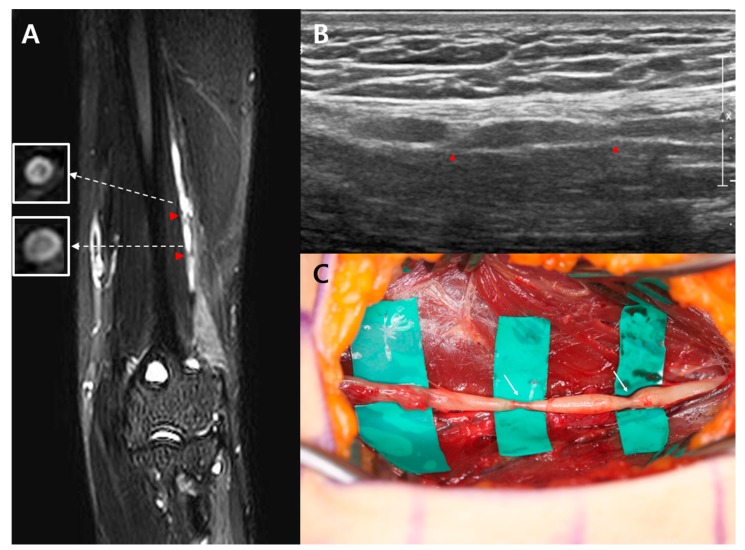
Case 2: (**A**) High-resolution magnetic resonance neurography reveals two focal constrictions (red arrowhead) of the left radial nerve 8.0 and 6.9 cm proximal to the lateral epicondyle, respectively. A bull’s eye sign (in white square) is observed juxta-proximal to each constriction site of the left radial nerve; (**B**) Longitudinal ultrasound reveals two constrictions (red arrowheads) of the left radial nerve; (**C**) Intraoperative findings confirm two constrictions (write arrow) of the left radial nerve.

A 19-year-old man visited the department of physical medicine and rehabilitation at Samsung medical center because of a sudden onset of left wrist drop and sensory deficits in the dorsum of the left hand and wrist. A day before the onset of the wrist drop, he had experienced diffuse pain around the left elbow joint and posterior forearm that had persisted for a day. The symptom onset was spontaneous, with no identifiable cause, 5 months before presentation. A physical examination revealed complete paralysis of the left wrist and finger extensors and hypoalgesia, and hypoesthesia at the distal borders of the forearm and hand. An MRI, electrodiagnostic study, and ultrasound were performed 5 months after the symptom onset. A cervical MRI revealed no abnormalities. On NCS, the CMAP of the left radial nerve showed a lower amplitude compared to the right radial nerve (left side: 0.6 mV vs. right side: 8.7 mV). On needle EMG, positive sharp waves were generated in the left brachioradialis, extensor carpi radialis longus, extensor digitorum communis, and extensor indicis muscles. These muscles showed no MUAP during volitional activity. The NCS and EMG findings indicated radial neuropathy between the mid-humerus level and the elbow joint. A high-resolution 3-Tesla MRN, performed with the same protocol as case 1, revealed two focal constrictions of the left radial nerve 8.0 and 6.9 cm proximal to the lateral epicondyle, respectively, and distal to the radial groove. Additionally, the bull’s eye sign was observed juxta-proximal to the constrictions of the left radial nerve. A longitudinal ultrasound revealed two nerve constrictions in the areas corresponding to the constrictions observed on MRN (Figure 2B). Approximately 6 months after onset, the patient underwent surgery for interfascicular neurolysis. The intraoperative findings confirmed constriction, as observed on MRN, 8.0 cm proximal to the lateral epicondyle (Figure 2C). The second constriction site was also seen. However, at the 6-month postoperative follow-up, the patient’s motor weakness showed no improvement.

## 3. Discussion

Here, we have described two cases of hourglass-like constriction neuropathy in the radial nerve, detected by MRN.

Largely, radial nerve palsy can be classified into compressive and non-compressive palsy [10]. Representative non-compressive causes include idiopathic neuralgic amyotrophy and hourglass-like constriction neuropathy [1]. The typical manifestation of idiopathic neuralgic amyotrophy is severe pain followed by weakness in one or more peripheral nerves [11,12]. Because hourglass-like constriction neuropathy can manifest as symptoms similar to those of idiopathic neuralgic amyotrophy, the differentiation of these two disorders is nearly impossible [1,11,12]. For diagnosing idiopathic neuralgic amyotrophy, other non-compressive or compressive causes should be ruled out. However, hourglass-like constriction neuropathy is often neglected in clinical practice, and its diagnosis is difficult. Therefore, many cases of hourglass-like constriction neuropathy are misdiagnosed as those of idiopathic neuralgic amyotrophy. Our patients could have been erroneously diagnosed with idiopathic neuralgic amyotrophy if hourglass-like constriction neuropathy was not suspected. A high-resolution MRN can detect hourglass-like constriction neuropathy of individual nerves, and we found hourglass-like constrictions of the radial nerves proximal to the elbow in both our patients. Further, in our patients, the bull’s eye sign was observed in the involved nerves. Although it has a low sensitivity, the bull’s eye sign is helpful for diagnosing hourglass-like constriction neuropathy [9]. To confirm the diagnosis of idiopathic neuralgic amyotrophy, all findings of hourglass-like constriction neuropathy should be ruled out.

The current use of the term “idiopathic neuralgic amyotrophy” or “hour-glass constriction neuropathy” is confusing. The term “hour-glass constriction neuropathy” is occasionally used to describe the phenomenon of idiopathic neuralgic amyotrophy, but both terms are also used separately. Although the pathophysiology of idiopathic neuralgic amyotrophy is largely unknown, the disease has been hypothesized to be an immune-mediated response to an unknown trigger. Before the era of high-resolution MRN or ultrasound, clinicians tended to diagnose idiopathic neuralgic amyotrophy as the cause of weakness, when the cervical spine or brachial plexus MRI did not show the cause of limb weakness. As new techniques in the field of medical imaging can demonstrate anatomic changes in detail, many patients previously diagnosed with idiopathic neuralgic amyotrophy are now diagnosed with hourglass-like constriction neuropathy. A recent study revealed a high incidence of focal hourglass-like constrictions (32/38 nerves) in 27 patients clinically diagnosed with idiopathic neuralgic amyotrophy [9]. The treatment may depend on the presence or absence of hour-glass constriction in patients clinically diagnosed with idiopathic neuralgic amyotrophy. Owing to these backgrounds, the definition or classification of the term “idiopathic neuralgic amyotrophy” or “hour-glass constriction neuropathy” should be re-established in future.

In addition, MRN is useful for finding the exact location of the nerve lesion. When the lesion of the radial nerve exists between the mid-humerus level and the elbow, electrodiagnostic study can detect whether the nerve lesion exists or not without providing the exact location, while MRN provides the exact location. Therefore, when surgery is required, the incision area can be reduced after MRN evaluation.

Other than high-resolution MRN, high-resolution ultrasound is also reportedly helpful for finding constrictions of the peripheral nerves and diagnosing hourglass-like constriction neuropathy [1]. Several previous studies have reported ultrasound findings of hourglass-like constriction neuropathy or neuralgic amyotrophy (Table 1) [4,5,13,14,15]. In these studies, various ultrasound findings, including focal or diffuse nerve/fascicle enlargement, nerve constriction, and fascicular entwinement, were shown. In 2017, Arányi et al. found that the sensitivity of ultrasound for diagnosing hourglass-like constriction neuropathy or neuralgic amyotrophy was 74% [5]. Ultrasound findings of our patients also revealed constrictions of the radial nerves. However, the reliability of ultrasound for diagnosing hourglass-like constriction neuropathy should be further explored. Therefore, for a more accurate diagnosis, a combined evaluation using high-resolution MRN and ultrasound is necessary.

Before the development of high-resolution MRN and ultrasound, hourglass-like constriction neuropathy was only diagnosed using exploratory surgery. In 2009, Vigasio et al. reported that preoperative differential diagnosis of hourglass-like constriction neuropathy and idiopathic neuralgic amyotrophy was unreliable [16]. Regarding the usefulness of MRN for detecting hourglass-like constriction neuropathy, a few studies reported findings of hourglass-like constriction in the brachial plexus and suprascapular nerve, which were detected by MRN (Table 1) [1,6,9]. However, to date, many clinicians are unaware of the usefulness and necessity of MRN for diagnosing hourglass-like constriction neuropathy or idiopathic neuralgic amyotrophy.

There was an apparent discrepancy between clinical manifestations and imaging. Patient 1 had no sensory deficit, whereas Patient 2 had sensory deficits in the dorsum of the left hand. This phenomenon may be explained by the concept of peripheral nerve topography. A recent study revealed that of 19 patients with clinical diagnosis of posterior interosseous nerve syndrome, 84% had lesions at the upper arm level instead of forearm level [17]. Clinical manifestations may vary depending on the severity of fascicular constrictions within the epineurium at the upper arm level.

The prognosis in our cases was poor, regardless of surgical treatment. Surgery is indicated for compressive or constriction lesions and for failure of conservative treatment [18]. The poor prognostic factors include age above 50 years, delayed surgery, and severe fascicular thinning [18]. Although the follow-up of 6 months was relatively short, the patients’ poor prognosis was probably related to severe constrictions. Future large-scale studies should clarify the role of surgical treatment on patients with severe constrictions.

## 4. Conclusions

In the current study, we described two cases of hourglass-like constriction neuropathy in the radial nerve, detected using high-resolution MRN. MRN is a useful tool for diagnosing hourglass-like constriction neuropathy and finding the exact lesion location. Our study is limited in that it is a case report. Studies involving a large number of cases should be conducted. Furthermore, the reliability and validity tests of MRN should be conducted in the future.

## Figures and Tables

**Table 1:** Summary of the previous studies on findings of magnetic resonance neurography or ultrasound of hourglass-like constriction neuropathy or neuralgic amyotrophy.

First Author, Year	Evaluation Tool	Summary
Van Rosmalen, 2019 [15]	US	51 patients with NA (upper limb) vs. 50 control subjects Increased cross-sectional areas in the affected nerves.
Kim, 2019 [1]	MRN	2 patients with SSN and 1 patient with SSN + RN Single focal constriction, multiple focal constrictions, diffuse swelling, and increased signal intensity
Sneag, 2018 [9]	MRN	27 patients with NA in the brachial plexus Focal intrinsic constrictions (32 of 38 nerves) Bull’s eye sign
Sneag, 2017 [6]	MRN	6 patients with NA SSN, AN, RN, AIN
Arányi, 2017 [5]	US	53 patients with NA with 70 affected nerves AIN (23%), RN (17%), LTN (17%), SSN (11%), accessory nerve (9%), and AN (7%) Sensitivity of US: 74% Swelling without constriction, incomplete constriction, complete constriction
Noda, 2017 [14]	US	6 patients with segmental swelling (larger cross-sectional diameter) Involved nerves: LTN, SN, MN, MCN, and PIN
Lieba-samal, 2016 [13]	US	4 patients with distal NA in AIN Swelling or hypertrophy of the involved nerves.
Arányi, 2015 [4]	US	14 patients with NA RN, PIN, AIN, SSN, MCN, LTN, MCN, and AN Four types of abnormalities: (1) focal or diffuse nerve/fascicle enlargement; (2) incomplete nerve constriction; (3) complete nerve constriction with torsion (hourglass-like appearance); and (4) fascicular entwinement. The types of abnormalities had no correlation with the prognosis.

Abbreviations: US, ultrasound; MRN, magnetic resonance neurography; NA, neuralgic amyotrophy; SSN, suprascapular nerve; AN, axillary nerve; RN, radial nerve; AIN, anterior interosseous nerve; LTN, long thoracic nerve; MN, median nerve; MCN, musculocutaneous nerve; PIN, posterior interosseous nerve.
